# A Case of Tracheotomy for Removal of a Foreign Body

**Published:** 1890-02

**Authors:** R. O. Cotter, H. J. Williams

**Affiliations:** Macon, Ga.; Macon, Ga.


### A CASE OF TRACHEOTOMY FOR REMOVAL OF A FOREIGN BODY.

BY R. O. COTTER, M. D., AND H. J. WILLIAMS, M. D., MACON, GA.

On the 27th of November the patient, a stout, healthy child of sixteen months, was brought to the reporter from Dodge county. Its parents stated that on the 23rd, while the child was playing in a pile of shelled corn in a room, it suddenly began to cough violently and choke up—all the symptoms of its having a foreign body in its wind-pipe were plainly apparent. They tried the very rational plan of causing the child to vomit, but without relief. The child grew worse. Its breathing becoming more and more difficult, and by the morning of the 27th, (the day when Dr. H. J. Williams and I first saw, and H. J. Williams, operated upon it), it could not rest except when lying almost upright in its mother's arms. Its breathing was very labored, and its face was becoming alarmingly cyanotic. While we could not positively distinguish the movements of a foreign body within the trachea, (by the way, I, for one, have never been able to positively do this in any of the cases I have seen), yet there were other pretty positive indications of there being such in this case. The seat of the trouble appeared to be just below the vocal chords, and dangerous laryngeal stenosis was rapidly supervening.

The child having been etherized tracheotomy was performed by Dr. H. J. Williams. As the child was quite fat, the operation was quite a tedious one. Only one blood vessel (a vein) was cut and ligated. More than once during the operation the child became seriously cyanosed; in fact, just before the opening was made in the trachea its condition was alarming. When the trachea was opened, instead of the foreign body being rapidly expelled, as they fortunately often are, to our great disappointment we found nothing. We supposed then that it must be a sharp, thin husk lodged somewhere about the glottis. The wound was allowed to remain entirely open, as we hoped that the foreign substance would sooner or later be forced out of it, and at any rate we noticed that the child's symptoms became decidedly better after the operation, although there still remained serious stenosis about the larnyx. For obvious reasons also, we, of course, did not insert a cavula.

On the evening of the 3rd day after the operation, on account of the slight bronchial irritation from the wound in the trachea, the child had some cough, and during one of its spells of coughing, to our great joy, it coughed out a small substance which careful examination showed to be a piece of a grain of corn, about the size of one-fourth of one's little finger nail. The size and shape of the substance had prevented its being blown out of the tracheal wound at the time of the operation.

The child improved rapidly after this, and as there was slight suppuration from the wound, and we could not hope for union by first intention, from sewing it up, we preferred to let it heal by granulation.

Tracheotomy undoubtedly saved the child's life, because, at the time of its performance the patient was rapidly sinking from laryngeal stenosis. The result shows that although the foreign body did not pass out through the artificial opening as they frequently do, opening of the trachea sustained the patient until the substance could be expelled by the natural channel.

On December 3rd the child was well enough to be sent home. The wound had almost closed. Last week (Dec. 8th) the father wrote that it was doing very well, though it was troubled with (bronchial) cough to some extent.

